# The Benefits of Whole-Exome Sequencing in the Differential Diagnosis of Hypophosphatasia

**DOI:** 10.3390/ijms252111728

**Published:** 2024-10-31

**Authors:** Oleg S. Glotov, Natalya A. Zhuchenko, Maria S. Balashova, Aleksandra N. Raspopova, Victoria V. Tsai, Alexandr N. Chernov, Iana V. Chuiko, Lavrentii G. Danilov, Lyudmila D. Morozova, Andrey S. Glotov

**Affiliations:** 1 Department of Genomic Medicine, D. O. Ott Research Institute of Obstetrics, Gynecology and Reproductology, 199034 Saint Petersburg, Russia; balashova_m_s@staff.sechenov.ru (M.S.B.); viktoriya14054@gmail.com (V.V.T.); anglotov@mail.ru (A.S.G.); 2 Department of Experimental Medical Virology, Molecular Genetics and Biobanking of Pediatric Research and Clinical Center for Infectious Diseases, 197022 Saint Petersburg, Russia; 3 CerbaLab Ltd., 199106 Saint Petersburg, Russia; raspopova_20@mail.ru (A.N.R.); i.danilov@spbu.ru (L.G.D.); 4 Department of Medical Genetics, N.V. Sklifosovsky ICM, I.M. Sechenov First Moscow State Medical University, 119991 Moscow, Russia; zhuchenko_n_a@staff.sechenov.ru (N.A.Z.); morozova_l_d@staff.sechenov.ru (L.D.M.); 5 Department of General Pathology and Pathological Physiology, Institute of Experimental Medicine, 197022 Saint Petersburg, Russia; 6 Department of Biological Chemistry, Federal State Budgetary Educational Institution of Higher Education Saint Petersburg State Pediatric Medical University of the Ministry of Health of Russia, 194100 Saint Petersburg, Russia; 7 Faculty of Bioengineering and Bioinformatics, Moscow State University, 119991 Moscow, Russia; mareckawert@gmail.com; 8 Department of Genetics and Biotechnology, Saint-Petersburg State University, 199034 Saint Petersburg, Russia

**Keywords:** hypophosphatasemia, whole-exome sequencing, differential diagnosis, phenotypic overlaps, rare diseases, *ALPL* gene pathogenic variants, clinical cases

## Abstract

Hypophosphatasia (HPP) is a rare inherited disorder characterized by the decreased activity of tissue-nonspecific alkaline phosphatase (TNSALP), caused by mutations in the *ALPL* gene. The aim of this study was to conduct differential diagnostics in HPP patients using whole-exome sequencing (WES). The medical records of HPP patients and the genetic testing of the *ALPL* gene were reviewed. Seven patients were recruited and underwent WES using the Illumina or MGI sequencing platforms. All of the exome samples were matched onto a GRCh38.p13 reference genome assembly by using the Genome Analysis ToolKit (GATK) and the BWA MEM read aligner. We present the clinical and molecular findings of the seven patients referred for genetic analyses due to a clinical and biochemical suspicion of HPP. In two patients out of three (with identified heterozygous variants in the *ALPL* gene), we also identified c.682T>A in exon 3 of the *WNT10A* gene and c.3470del in exon 23 of the *SMC1A* gene variants for the first time. In four patients, variants in the *ALPL* gene were not detected, but WES allowed us to identify for the first time rare variants (c.5651A>C in exon 36 of the *TRIO* gene, c.880T>G in exon 6 of the *TRPV4* gene, c.32078-1G>T in intron 159 of the *TTN* gene, c.47720_47721del in exon 235 of the *TTN* gene, and c.1946G>A in exon 15 of the *SLC5A1* gene) and to conduct differential diagnostics with HPP. Using WES, for the first time, we demonstrate the possibility of early differential diagnostics in HPP patients with other rare genetic diseases.

## 1. Introduction

Hypophosphatasia (HPP, OMIM: 146300, 241500, 241510) is a rare inherited disorder that was first described in 1948 [1]. HPP is characterized by a broad variety of clinical presentations and forms of severity. Depending on the age at onset and the severity, HPP is classified in six clinical forms, ranging from the perinatal severe form to isolated odonto-HPP and mild manifestations in adults [2,3].

The prevalence of the HPP varies, depending on the form of the disease: severe cases have less prevalence than mild forms and also depend on the region where the study has been done. The propagation of HPP in the European population is between 1:100,000 and 1: 300,000 for severe and 1:6370 for moderate forms [4]. The birth prevalence of severe HPP in Japan is estimated at 1:150,000 according to the frequency of individuals who are homozygous for the pathogenic variant c.1559delT [5]. The prevalence of HPP in China is still unknown. Zhang Q. reported five cases of HPP in a Chinese population [6] For a Canadian population, the frequency of the perinatal form of HPP was 1:2500, according to the variant p. Gly334Asp [7]. Recently, the increased incidence of HPP mild forms (1:508) has been observed [8].

The main cause of HPP is the decreased activity of the tissue-specific alkaline phosphatase (TNSALP), which is a membrane-bound glycosylated enzyme that is involved in bone mineralization. This bone-specific TNSALP isoform comprises 507 amino acids and is formed by the alternative splicing of the alkaline phosphatase (*ALPL*) gene, utilizing one of two leading exons [9]. In turn, the decrease in TNSALP activity is caused by the accumulation of mutations in the *ALPL* gene, which is located in chromosome 1p36.12 and consists of 12 exons [9]. For the first time in 1988, a pathogenic variant (c. 711A>G, Ala162Thr) in the *ALPL* gene was identified [10]. The global prevalence of pathogenic variants in the *ALPL* gene stands at 0.7% (7 cases per 1000), whereas its frequency in the Russian population is about 4.2% [11,12].

HPP is characterized by an autosomal recessive and a dominant inheritance pattern. If a mutation is detected in one allele, the clinical manifestations are mild, and the disease is expected to show an autosomal dominant heritage character with variable expression and incomplete penetrance. The presentation of HPP can be extremely variable within and between families, in both children and adults.

The accurate diagnosis of patients with a clinical suspicion of HPP is crucial, as not only is the disease life-threatening, but the patients may be offered bone-targeted enzymatic replacement therapy [13]. A HPP diagnosis is based on biochemical testing results (TNSALP serum activity), radiography findings, clinical symptoms, and mutations in the *ALPL* gene. A mandatory diagnostic criterion for HPP is low TNSALP activity, depending on age and gender [14]. TNSALP dephosphorylates many substrates, including inorganic pyrophosphate (PPi), pyridoxal-phosphate (PLP), and phosphoethanalomine (PEA) [15]. HPP patients tend to have a similarly elevated substrates pattern, and these molecules are used in HPP diagnoses. The International Working Group on HPP proposes to identify the primary and secondary criteria for HPP diagnoses. The main criteria include the presence of (1) *ALPL* gene variant(s); (2) an increase in TNSALP substrates; and (3) the early non-traumatic loss of primary teeth [16,17].

Low TNSALP levels require the further performance of differential diagnostics to exclude other alternative diseases that may contribute to the decreased activity of this enzyme (osteogenesis imperfecta) [17,18]. Genetic states, when a HPP patient has more than one monogenic disease, could cause complex clinical manifestations, which may have overlapping or different (composite) phenotypes [19]. Also, *ALPL* gene screening in HPP patients may be negative, indicating that clinical and biochemical parameters are not associated with this gene.

HPP diagnostics is a complex clinical problem that is associated with the absence of a “gold standard” in diagnosis, clinical heterogeneity, overlapping phenotypes, and the absence of significant variants in the *ALPL* gene. *ALPL* gene sequencing alone does not provide enough evidence to confirm or exclude pathological variants in other genes that may affect clinical manifestations. Today, clinicians are increasingly relying on whole-exome sequencing (WES), which is gaining prominence as a first-line diagnostic procedure, especially in metabolic and neurodevelopmental disorders in children who are presented for diagnostic investigation [20]. The aim of this study was to conduct differential diagnostics in HPP patients using WES.

## 2. Results

### 2.1. Molecular Doking of TNSALP

**Figure 1:**
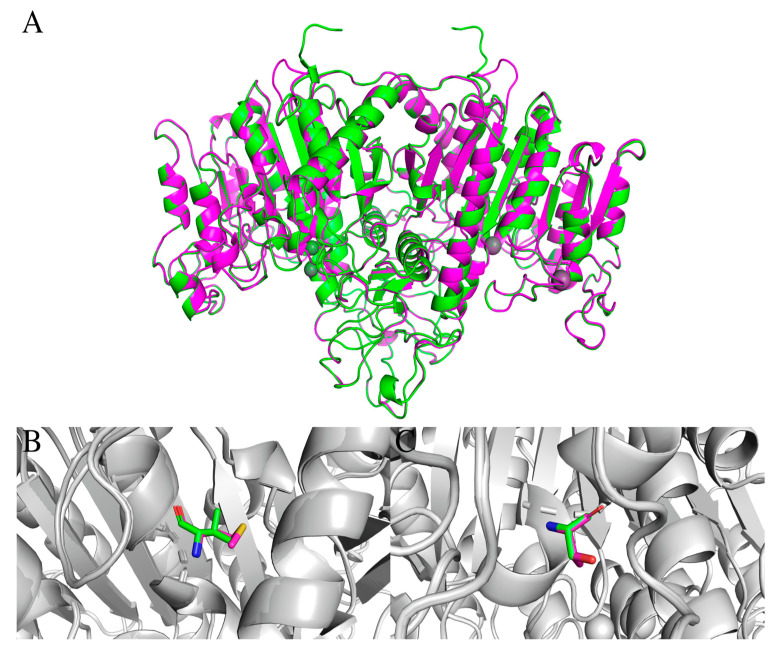
(**A**) Comparison of the structure of the wild-type protein, with Val483M and Ala69Thr mutant TNSALP proteins (WT—green color; mutated—pink color). (**B**) Val483Met replacement. (**C**) Ala69Thr replacement.

The TNSALP protein encoded by the *ALPL* gene is a dimer, in which each of the monomers appears as beta sheets surrounded by clusters of alpha helices (Figure 1A). The N-terminus of each monomer in the complex is formed by an alpha-helix that continues into the neighboring protomer in order to maintain the structure of the dimer. Amino acid residues, R71-D458, are important for maintaining the dimer’s structure through hydrogen bonding and various hydrophobic interactions. The TNSALP protein also contains catalytic binding sites that contain calcium, magnesium, and zinc ions. The amino acid residues in the catalytic center of the protein responsible for binding are located at R223-D306. Apparently, the detected V383M and A69T mutations (Figure 1B,C) can disrupt the structure of the TNSALP complex dimer.

**Figure 2:**
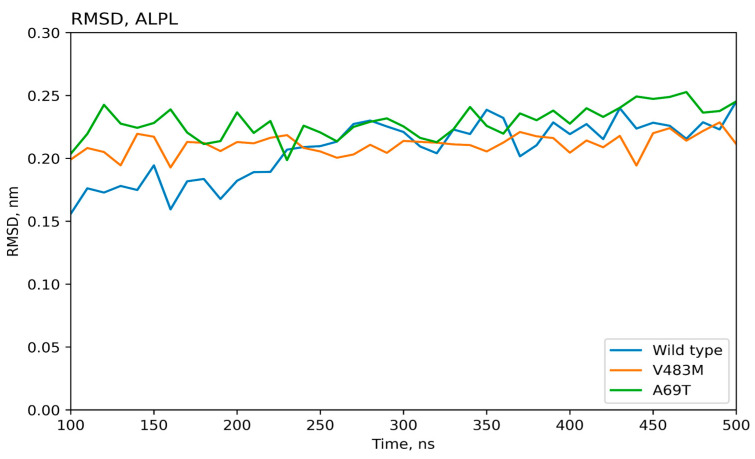
Time-dependent dynamics of atomic oscillations for wild-type and mutated (Val483Met, Ala69Thr) TNSALP proteins. RMSD—root mean square deviation.

**Figure 3:**
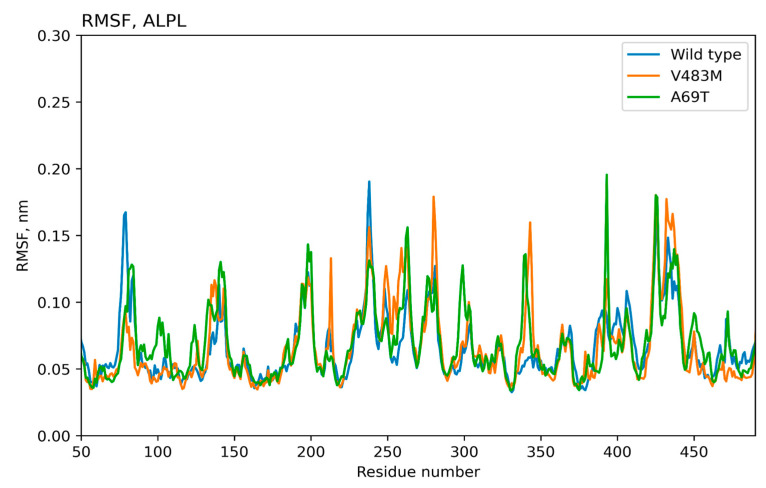
Time-dependent dynamics of amino acid residue fluctuations for wild-type and mutated (Val483Met, Ala69Thr) TNSALP proteins. RMSF—root mean square fluctuation.

The molecular dynamics results for 500 ns were used to obtain atomic standard deviations and root mean square (RMS) fluctuation values for the wild-type protein and proteins with introduced mutations, Figure 2 and Figure 3. Thus, the stability of the protein structure can be observed during the molecular simulation time. As can be seen, the values of the RMS deviation of atoms during the dynamics for all the variants of the TNSALP protein do not exceed the value of 3 angstroms. This indicates the complete stability of the dimer’s structure during 500 ns (proteins with RMS deviation not exceeding 3 angstroms are considered identical). These amino acid residues may be involved in the formation of a tetramer, another form of TNSALP.

### 2.2. Clinical and Genetic Characterization of HPP Patients

Our study describes seven clinical cases of patients who were presumptively diagnosed with HPP, based on clinical findings. All the individuals were presented with low TNSALP and developmental delay (growth retardation, various skeletal deformities, poor weight gain, muscle hypotonia, and other reported HPP-associated symptoms), Table 1.

At the outset, all the patients underwent Sanger sequencing, with heterozygous variants in the *ALPL* gene revealed in three of the patients. The purpose of the subsequent WES was to clarify the diagnoses, considering that in three of the patients, a heterozygous variant in the coding region and flanking regions of the *ALPL* gene failed to offer any adequate explanation of the symptoms. The cases without detected variants in the *ALPL* gene required further diagnostic efforts, i.e., performing a differential diagnosis to consider alternative diagnoses, Table 2 and Table 3.

Case 1. A 10-year-old female patient was born at term (body weight at birth 2895 g) to healthy, unrelated parents. At the age of 1.5 months, the first epileptic paroxysms were observed, which presented as impaired consciousness with wheezing, tonic–clonic seizures, and the upturning of the eyes. Until 4 years of age, the paroxysms were recurrent, occurring as a series of events two to three times per month; later, the patient deteriorated, with the paroxysmal events occurring three to eight times per day, while the seizures were controlled by large doses of pyridoxine. A brain MRI revealed mild external hydrocephalus. After studies were conducted, within the first 2 years, the child was excluded from having hereditary metabolic diseases, clinically significant microdeletions, or Rett syndrome. Since early childhood, the patient exhibited developmental retardation, with her body weight being 14.5 kg (percentile 3.7) at the age of 10 years. Her growth was reduced, and she also exhibited mental, speech, and motor retardation (she was unable to sit, stand, or walk). Low TNSALP levels were first noted at almost 2 years of age, but this did not alert the doctors. At age 5, due to a deterioration in general health, the patient was re-examined, which revealed a significant decrease in TSALP, to 69–73 IU/L (reference 150–370 IU/L), although calcium, ionized calcium, vitamin D, and phosphorus were within normal limits; elevated levels of TSALP substrates were also detected: PPi and PEA, Table 1.

Sequencing of the *ALPL* gene revealed *c.1447G>A* in exon 12 (chr1:g.21904013G>A; rs1256212515), a heterozygous variant. At the age of 6 y., based on clinical and genetic findings, the patient was diagnosed with infantile HPP and approved for asfotase-alpha enzyme replacement therapy. Over the course of 3 years of asfotase alfa enzyme replacement therapy, the convulsive paroxysms decreased in frequency to one episode in 3 weeks. However, the patient’s body weight deficit and psychomotor retardation persisted. Seizure-related fractures of tubular bones were reported three times, with the persistent pronounced osteoporosis of tubular bones, assessed by radiographic imaging. Considering the disease’s progression and resistance to therapy, the patient underwent WES. Exome sequencing confirmed the *c.1447G>A* heterozygous variant in the *ALPL* gene. The patient also carried a heterozygous *c.3470del* in exon 23 (chrX:g.53407975CT>C) variant in the solute carrier family five, member one (*SMC1A*) gene (OMIM: 300590; 301044), Table 2. The differential diagnostics and phenotypic overlaps are presented in Table 3.

Case 2. A 4 years old female patient, born at term, with a body length of 52 cm and a weight of 3300 g., manifested muscle hypotonia, growth retardation, poor body mass gain, and breath-holding spells since birth. Examination at the age of 4years revealed retarded physical development (height 94 cm (4 percentile, SDS growth = −1.6), body mass 14.5 kg (23 percentile, SDS BMI = −0.6)), planovalgus foot deformity, the loss of teeth, brittle hair, hypermetropia, and bilateral astigmatism. Blood chemistry showed persistent diminished TSALP and a moderately elevated phosphate concentration in urine. Radiography of the wrist showed diminished ossification, with the bone age corresponding to the age of 2.5–3 years), Table 1.

In this patient, we identified *c.1447G>A* in exon 12 (chr1:g.*21904013G>A*; rs1256212515), a heterozygous variant in the *ALPL* gene. The patient was also reported to have the *c.682T>A* (chr2:g.219755011T>A; rs121908120) homozygous variant in exon three of the Wnt family member 10A (*WNT10A*) gene (OMIM: 257980; 224750; 150400), Table 2. The differential diagnostics and phenotypic overlaps are presented in Table 3.

**Figure 4:**
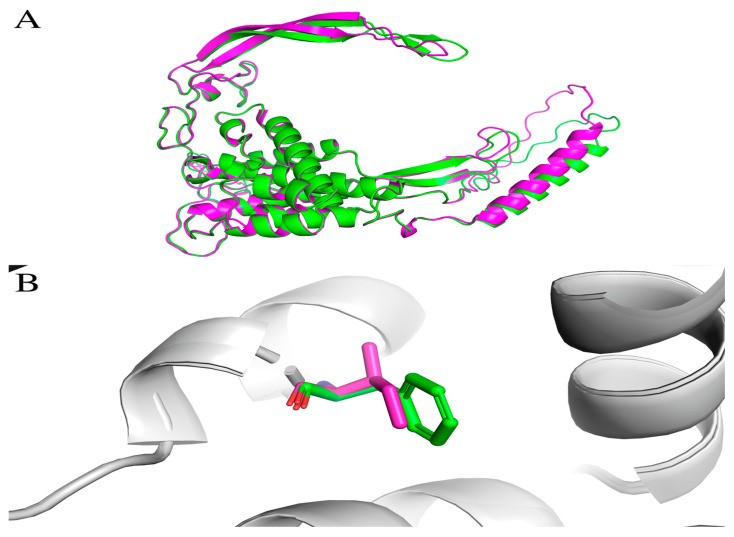
(**A**) Comparison of the protein structures of wild-type WNT10A with the Phe228Ile mutant (WT—green color, mutated—pink color). (**B**) Phe228Ile replacement.

We also conducted molecular docking for proteins encoded by the *WNT10A* gene, Figure 4A. A protein is a transmembrane receptor with a signaling function. Therefore, obtaining their exact experimental structure, as well as their behavior during molecular dynamics, is a challenging task. Nevertheless, the *Phe228Ile* mutation (Figure 4B) can also affect the stability of the protein’s structure, which was verified by molecular dynamics. It is known that the part of transmembrane proteins that is located directly between the bilipid layers of the cell wall is represented by often-extended alpha-helixes; the identified mutation is in one of them, from the protein *WNT10A***.**

**Figure 5:**
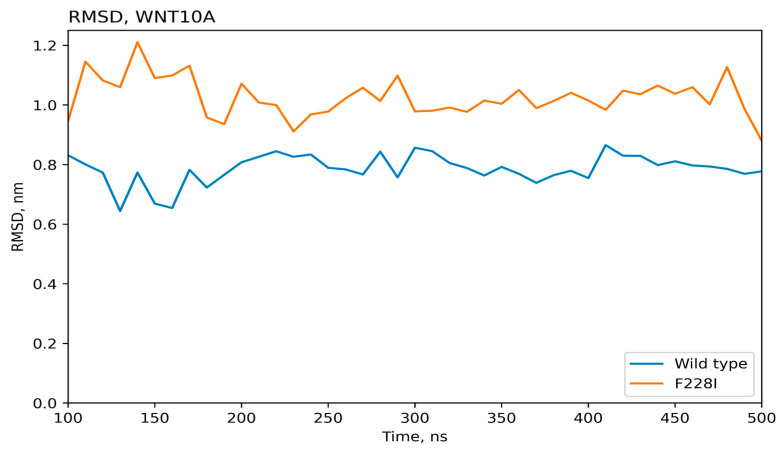
Time-dependent dynamics of atomic oscillations for wild-type and mutated (Phe228Ile) WNT10A proteins. RMSD—root mean square deviation.

**Figure 6:**
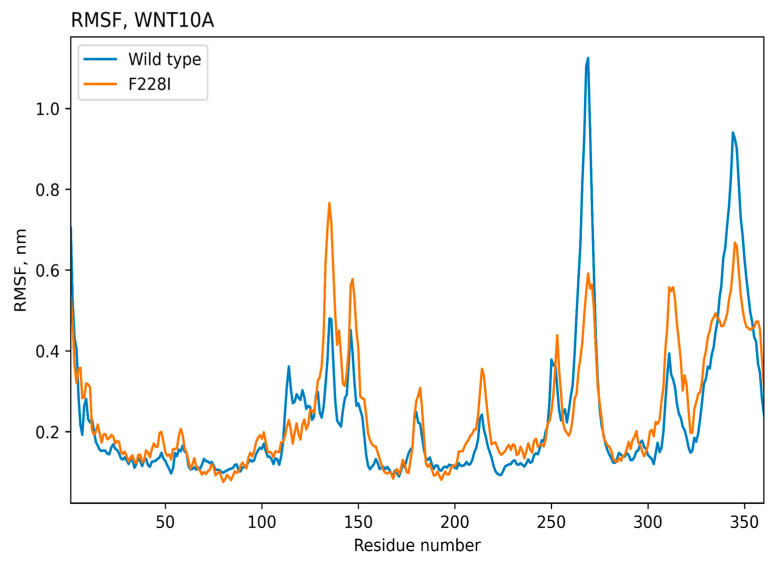
Time-dependent dynamics of amino acid residue fluctuations for wild-type and Phe228Ile -mutated WNT10A proteins. RMSF—root mean square fluctuation.

According to the results of molecular modeling, it turned out that the average standard deviation of atoms for the WNT10A protein is quite high (close to 10 angstroms), indicating the instability of the structure, Figure 5 and Figure 6. This is indeed characteristic of transmembrane proteins, but for the protein with the introduced *Phe228Ile* mutation, the values of the mean square deviations of atoms over time are even higher; hence, this mutation can disrupt the protein structure.

Case 3. A 14-year-old female patient, born at term, had a birth body length of 52 cm and a body weight of 4400 g. The child’s development progressed according to age norms. At 12 years old, the patient was diagnosed with micropsia and was assessed by an ophthalmologist. Since early childhood, the patient showed rapid weight gain. For the last two years, the patient has presented with a low TSALP level (43–81 IU/L). The patient reported pain in the left knee joint, hair loss, and excessive weight gain. On examination, the patient’s height was 172 cm (1.73 SDS), her weight was 91 kg, and her body mass index (BMI) was 30.76 kg/m^2^. Her bone structure was without deformities. Her dental formula corresponded to her age. Abdominal sonography showed liver enlargement, thickening of the gallbladder walls, dyscholia, and the bilateral diffuse involvement of the pelvicalyceal walls. Radiography of the wrists and hands displayed expressed regional osteoporosis without a bone age decrease. Radiography of the knee joints showed no destructive bone disease; the left tibial metaphysis displayed a round lucent region in the bone, 1.7 cm × 1.1 cm, with clear smooth margins (presumably a metaphyseal fibrous cortical defect or osteoid osteoma?), Table 1. The *ALPL* gene sequencing detected the *c.205G>A* in exon four (chr1:*21887613G>A*; rs1178008018), a heterozygous mutation. WES refinement did not yield any new results, Table 2.

Case 4. A 2-year-old male patient presented with muscle hypotonia, growth retardation, and poor body weight gain since birth. At the age of 2 years, an examination revealed physical development retardation (body weight 5 kg, height 69 cm), as well as expressed skeletal deformities, Table 1. The child had a retardation of their mental and motor development, manifesting as an inability to sit, stand or walk, as well as an absence of speech. Active and passive movements of the knee and ankle joints were constrained. Laboratory findings: a complete blood count, urinalysis, and the blood chemistry (including calcium and phosphorus) were within reference; decreased TSALP activity, to 85.2 IU/L (reference 150–370 IU/L), and decreased ionized calcium, to 0.99 mmol/L (reference 1.05–1.32 mmol/L), were identified. Electrocardiogram (ECG) revealed myocardial repolarization disorders. No other abnormalities were detected, Table 1. Sanger sequencing and WES did not detect any variants in the *ALPL* gene. Additionally, a heterozygous variant, *c.5651A>C* in the 36th exon (chr5:g.14463018A>C), was detected in the trio Rho guanine nucleotide exchange factor (*TRIO*) gene (OMIM: 617061; 618825). In addition, next-generation sequencing revealed a heterozygous variant (c.880T>G exon six (chr12:g.110236691A>C)) in the transient receptor potential cation channel, subfamily V, member four (*TRPV4*), gene (OMIM: 606071), Table 2. The differential diagnostics and phenotypic overlaps are presented in Table 3.

Case 5. A 12-month-old female patient, born with a normal body length and weight, presented with muscle hypotonia, motor development retardation, and skeletal deformities that had appeared immediately after birth. An examination revealed pectus excavatum and contractures of the wrist joints. The patient was remarkable for motor development retardation, had decreased muscle tone in the upper and lower limbs, and had constrained active movements (she was unable to turn over independently and unable to hold her head). Abdominal and renal ultrasound examinations showed no abnormalities. The lower reference limits of TSALP were recorded repeatedly, Table 1.

Sanger sequencing and WES did not detect any variants in the *ALPL* gene. However, heterozygous variants were found in the titin (*TTN*) gene: *c.32078-1G>T* in intron 159 (chr2:g.179516477C>A) and c.47720_47721del in exon 235 (chr2:g.179466391CTT>C) (OMIM: 611705; 608807), Table 2. The differential diagnostics and phenotypic overlaps are presented in Table 3.

Case 6. A 4.5-year-old female patient was born to a consanguineous couple, related as third cousin and sister. The child was born to a nulliparous mother in the second pregnancy, complicated by an intrauterine infection (the first pregnancy was terminated). The mother’s third pregnancy resulted in giving birth to a child who died at the age of 9 months. The patient was born at term by C-section, with a birth weight of 4100 g, a height of 52 cm, and an Apgar score of 2/5. At the age of 6 weeks, the patient presented with symptoms that were indicative of gastroenteritis, including poor weight gain, mild anemia, and marked hypotrophy and muscle hypotonia. Renal sonography revealed kidney calcification. Early psychomotor development showed marked retardation. At the age of 6 m., the patient underwent transcranial magnetic stimulation (TMS), with no evidence of hereditary aminoacidopathies, organic aciduria, or mitochondrial beta-oxidation defects. At the age of 13 months, the first evidence of a decreased TSALP level, to 139 IU/L (reference 150–350 IU/L), was obtained by blood chemistry. An examination at the age of 4.5 years displayed a height of 95 cm (SDS height—3) and a weight of 14.5 kg (SDS weight—1). The patient’s phenotype featured a smoothed philtrum, thin lower lip, and larger and slightly deformed auricles; the patient showed partial adentia: two of the anterior incisors of maxillar were absent, with the destruction of neighboring incisors (the change of dentition started at 2 years of age, with the loss of the primary molars). Chest deformity, a distended abdomen, posture disorders, winged scapula, spinal hyperkyphosis, a valgus deformity of the lower limbs, a planovalgus alteration of the feet, muscular hypotonia, and severe psychomotor development retardation were identified. Blood chemistry findings: decreased TSALP level (118 IU/L, reference 156–369 IU/L), Table 1.

Sanger sequencing and WES did not detect any variants in the *ALPL* gene. WES revealed a homozygous variant, c.1946G>A, in exon 15 (chr22:g.32506151G>A) in the *SLC5A1* gene (OMIM: 606824), Table 2. The differential diagnostics and phenotypic overlaps are presented in Table 3. Considering the WES results, the diagnosis of glucose–galactose malabsorption was assumed. Dietary nutrition is recommended, with the elimination of lactose, glucose, and sucrose from diet.

Case 7. A 3-years-and-9-months-old male patient was born at term (38 weeks), following the third pregnancy to a nulliparous mother (two earlier pregnancies resulted in stillbirth), by C-section delivery. His birth body weight was 1640 g, and his birth body length was 40 cm. From birth, the boy showed growth retardation and poor body weight gain. At 9 months, his body weight was 5900 g, and his body length was 55 cm; muscle hypotonia was reported. At the age of 18 months, the child continued to display pronounced developmental retardation, exacerbated by mental and speech retardation and rumination syndrome. A complete blood count revealed iron deficiency anemia (hemoglobin, Hb 90 g/L); the TSALP level was at the lower threshold of the reference range. An examination at the age of 3 years and 9 months revealed stunted growth (height 74 cm, SDS height—5.51), a severe protein-energy deficit (weight 5.86 kg, SDS BMI-4.83), severe mental and speech disorders, and musculoskeletal abnormalities: chest asymmetry, flat feet, moderately reduced motor activity, and muscle hypotonia in the upper and lower extremities. His teeth were demineralized, with scattered areas of destruction. Phenotypic manifestations included small facial features, blepharophimosis, epicanthus, a smoothed lip groove, a thin upper lip, and micrognathia. Abdominal and renal sonography revealed a kinking of the gallbladder, right-side pyelectasis, and hydrocalycosis with incomplete bilateral pelvicalyceal duplication. Cardiac sonography showed a patent foramen ovale. Other findings included OU-hypoplasia, partial optic atrophy, secondary high myopia, and left eye strabismus (Table 1). Blood chemistry findings showed a TSALP level of 126 IU/L (reference 125–320 IU/L), at the lower threshold of the reference. Sanger sequencing and WES did not detect any variants in the *ALPL* gene or other genes. Deep phenotyping established fetal alcohol syndrome.

## 3. Discussion

HPP is a genetic disorder of bone metabolism, featuring a variegated clinical presentation. The clinical spectrum ranges from severe forms with extreme skeletal deformities, respiratory impairment, and seizures, to very mild forms with an onset in late adulthood and few clinical signs [21]. The diverse clinical manifestations of HPP often lead to erroneous diagnoses, the misinterpretation of signs, and delays in the correct diagnosis. Global HPP Registry indicates that the median time between the onset of symptoms and the diagnosis of HPP is 5.7 years. [22].

Our study included patients with tentatively diagnosed HPP, based on clinical findings (low TSALP, growth retardation, poor weight gain, various skeletal deformities, muscular hypotonia, and other symptoms). However, despite the presence in the seven cases of a number of similar clinical manifestations that overlap with the symptom complex of hypophosphatasia, very different results were obtained by WES. In one case, genetic conditions were identified (a combination of hypophosphatasia and epileptic encephalopathy). In Case 2, the variant described for hypophosphatasia was identified, and a variant of uncertain significance was additionally identified in the *WNT10A* gene. In Case 3, the only finding through WES was a variant of uncertain significance in the *ALPL* gene, which did not explain the cause of the disease. In Case 4, two VUSs were detected in the *TRIO* and *TRPV4* genes, potentially associated with the clinical picture, but requiring additional examination. In Cases 5 and 6, variants were found that indicated other hereditary diseases (titinopathy and glucose–galactose malabsorption). In Case 7, no candidate genetic variants were found, but signs of fetal alcohol syndrome were identified. These clinical cases are considered to involve both the known issues of HPP differential diagnostics and new ones arising from the use of next-generation sequencing (NGS) technologies: biochemical parameters; the presence of variants in the *ALPL* gene and other genes in the genotype; and the absence of variants in the *ALPL* gene.

### 3.1. Biochemical Parameters

Biochemical parameters play a fundamental role in any diagnostic process. The measurement of TSALP activity is a distinctive and mandatory feature for HPP diagnostics [9,23]. Repeated blood samplings are necessary to confirm persistently subnormal TSALP activity, as a single blood test is not sufficient to diagnose or exclude HPP. In our study, almost all of the patients had persistently low TSALP activity, Table 1. In a clinical routine, lowered TSALP values are often overlooked, which makes the diagnosis of HPP difficult and often leads to pronounced delays in therapy, which was also observed in our cases. Thus, in the first case, a TSALP low level was first noted at almost 2 years of age, but this indicator did not alert the doctors.

However, it is important to stress that low TSALP values are not synonymous with HPP; therefore, the assessment of patients with reduced TSALP activity requires the exclusion of other pathologies that may be associated with this alteration [17,24]. Table 4 summarizes the main conditions that should be considered in the differential diagnosis of HPP.

The causes of low TSALP levels include malignancies, endocrine or metabolic disorders, inflammatory and renal diseases, nutritional deficiencies, and drugs. Divalent ions of Mg^2+^, Co^2+^, and Mn^2+^ are activators of TSALP, and Zn^2+^ is a constituent ion of the enzyme. Zn^2+^ deficiency has been shown to reduce the activity of bone-related enzymes (TSALP) and minerals (Ca^2+^, P and Mg^2+^) [25]. Malnutrition causes a deficiency of the proteins, vitamins, minerals, and nutrients essential for the synthesis and proper functioning of TSALP. An inadequate intake of these nutrients can impair the enzyme’s production and activity. Finally, intestinal damage or inflammation and protein-energy malnutrition can reduce ALP production, leading to lower TSALP levels [26].

Among the presented clinical cases, three patients (4, 6, 7) were found to have low TSALP levels and protein-energy malnutrition, Table 1. In one patient (Case 6), WES revealed a rare inherited disorder of glucose–galactose malabsorption, which was accompanied by bouts of diarrhea and led to severe malnutrition. In another patient (Case 7), the use of “deep phenotyping” revealed fetal alcohol syndrome; children with PAE may be at risk of multiple nutrient deficiencies [27]. It is also known that any disorder which impairs linear growth has the potential to result in lower serum TSALP activity, since markers of bone turnover are indeed influenced by linear growth in children [16,28]. Linear growth was disrupted in five of the patients (Cases 1, 2, 4, 6, and 7), namely in the HPP patients with glucose–galactose malabsorption and with fetal alcohol syndrome, Table 1.

In addition to the known causes, low TNSALP levels may be associated with less common situations and sometimes rare diseases that are also associated with low TNSALP levels, which should be excluded using differential diagnostics. Diseases such as cleidocranial dysplasia, Mseleni joint disease, osteogenesis imperfecta, and Wilson’s disease are associated with low TNSALP activity [29,30]. Subnormal enzyme levels have been observed in 60–90% of individuals with Wilson’s disease, primarily in patients with severely impaired hepatic function; however, TNSALP levels are not specific to this condition. Some researchers suggest that zinc deficiency may be involved [31]. Recent studies have shown that iron and ferritin are potent inhibitors of osteogenesis, significantly inhibiting TNSALP activity in hemochromatosis. However, it is not currently included in the differential diagnosis of HPP. The mechanism of the development of low TNSALP levels in many diseases is uncertain. We have not been able to find out whether a low TNSALP level is typical for TRPV4 disorders, Case 4. However, experimental data on cell lines show that *TRPV4* mutations suppress the expression of the *ALPL* gene, which theoretically can lead to a decrease in TNSALP activity [32]. Furthermore, TNSALP has a calcium domain in its structure, so a mutation in the *TRPV4* gene can influence TNSALP activity. Knowing the causes of low TNSALP levels is of great importance when it comes to properly diagnosing the disease.

The clinical manifestations of HPP are due to the low TNSALP activity that leads to the accumulation of its substrates (PPi, PLP, PEA). To date, PPi measurements are not routinely performed, while PLP (vitamin B6) and PEA measurements are available in many laboratories. Recent studies show that PLP appears to have higher sensitivity and specificity compared to other substrates, such as PEA, for HPP diagnostics. Although the PLP serum level in HPP patients varies greatly, it is elevated in almost all cases [13,22]. One of the patients in this study (Case 1) had elevated PPi and PEA levels, and the HPP diagnosis was confirmed.

The detection frequency of mutations in the *ALPL* gene is higher in cohorts of patients with low TNSALP levels and high PLP levels (66.7%) than among patients with only low TNSALP levels (43%, 47%) [19,33]. The measurement of natural TNSALP substrates is recommended for the diagnosis of HPP, but also for differential diagnoses. The International Working Group on HPP proposes to include an increase in the natural substrates of TNSALP in the primary HPP criteria.

In clinical practice in different countries, as well as in Russia, low TNSALP levels are the most important parameter for diagnosing HPP, while the natural substrates of TNSALP are rarely measured [1,17].

### 3.2. The Presence of Variants in the ALPL Gene and Other Genes

Evidence suggests that clinical HPP symptoms and their severity can significantly vary among family members, as well as unrelated patients [8,12,30]. The high clinical heterogeneity is mainly due to the great number of *ALPL* missense mutations (about 74%), with more than 400 different mutations having been described [34,35]. An imperfect correlation between the genotype and the phenotype suggests that other genetic or environmental factors modulate the phenotype. Factors determining the clinical heterogeneity between patients with the same *ALPL* genotype include the presence of variants in other genes in the genotype (*COL1A2*, *COL1A1*, *RUNX2,* and others) [28,36].

This study describes two clinical cases with a heterozygous variant, *c.1447G>A* (*p.Val483Met*), in the *ALPL* gene (Cases 1 and 2). Whole-exome sequencing revealed variants in other genes in these patients as well, Table 2. The patient described in Case 1 was found to have a heterozygous variant, c.3470del, in the *SMC1A* gene (which encodes the chromosome structure maintenance of protein 1A, which is involved in cell division, the regulation of expression, and DNA repair). The dominant symptom in our patient was seizures, resembling those reported in developmental and epileptic encephalopathy 85 (DEE85) and characterized by cycles of drug-resistant epileptic paroxysms with clustering, as well as a global developmental delay [37]. Low TNSALP levels, increased PPi and PEA levels, rickets-like bone deformities, and osteoporosis are attested in HPP, but not in cohesinopathy disorders (associated with mutations in the *SMC1A* gene). On the one hand, epileptic paroxysms can be caused by the detected mutation in the *SMC1A* gene. In contrast, seizures can be associated with early onset of severe HPP. In our case, epileptic paroxysms appeared in the first 4 weeks after birth, with high doses of pyridoxine being administered for severe seizures. TNSALP hydrolyzes PLP, which is the major circulating form of vitamin B6, causing vitamin B6 deficiency in the central nervous system, which is apparently responsible for the onset of epileptic seizures [38]. Asfotase alpha therapy initiates the hydrolysis of Pi, thus reducing the amount of accumulated extracellular TNSALP substrates and improving skeletal mineralization [39]. In Case 1, asfotase alfa therapy did decrease the frequency of seizures, with no improvement of the developmental delay. Case 1 was initially diagnosed with HPP (infantile form), confirmed by a detected c.1447G>A heterozygous variant in the *ALPL* gene. The patient presented a few overlapping clinical features that were common for both HPP and DEE85: refractory seizures that manifest in the first year of birth and a developmental delay. WES revealed two rare monogenic diseases in one patient, Table 3.

In the second patient, we also identified a mutation in the *WNT10A* gene that encodes the WNT family member 10A protein that is involved in ectodermal tissue development and canonical Wnt signaling pathway activation, affecting ectodermal patterning. Case 2 presented with hardly any ectodermal pathology (except for brittle hair and tooth decay); muscle hypotonia, growth and developmental delay, respiratory attacks, and low blood TNSALP levels, though typical of HPP, have never been reported in relation to the detected *WNT10A* gene variant, and so this case requires further clinical follow-up. Some of the patients presented with a HPP pathognomonic manifestation, although the phenotype showed broad variability, Table 1.

The expanding use of NGS technology is increasing the number of patients who are found to have multiple disorders, some of which may have overlapping phenotypes, while others may be “incidental findings” [40]. The proportion of multiple disorders in a single patient has been estimated at approximately 2–7.5% of diagnosed cases, largely depending on the studied cohort [41]. The reported cases are a good illustration of how different genetic conditions and molecular mechanisms could combine in a single patient, causing peculiar and complex clinical pictures.

This study describes three clinical cases with heterozygous variants in the *ALPL* gene. In a Russian population, the heterozygous carriage of variants in the *ALPL* gene was found in 225 (13.9%) out of 1612 patients with reduced TNSALP activity [12]. Depending on the HPP form and the clinical symptoms identified among the Russian patients, an analysis of the *ALPL* gene variants showed that compound heterozygotes and heterozygotes were prevalent (*p* < 0.01) in patients with infantile and childhood HPP compared to perinatal HPP. In theory, heterozygotes have higher residual enzyme activity than homozygotes. The *c.1447G>A* variant (*p.Val483Met*) was detected in two cases. With HPP prevalence in the Russian population estimated at 1.11% (5/450), this variant was earlier described in Russian patients with childhood HPP [19]. According to the gnomAD database, the *c.1447G>A* variant has the frequency of 0.00001971 (to the date of last access). The replacement of valine by methionine (*p.Val483Met*) has been observed in an evolutionary conserved homodimeric domain. Such missense mutations can cause impaired homodimer formation with subsequent protein incorporation into the cell membrane [12]. The investigation of the *ALPL* gene variants and their activity in relation to mutation origin and mutation-harboring domains suggests that decreased (*p* < 0.05) TNSALP activity is characteristic of variants that encode amino acids at the active site and the crown domain (i.e., the protein regions that are critical for maintaining enzyme activity), rather than variants at the homodimer interface (non-specific protein regions). This conclusion is underpinned by the results of our earlier study, which attempted to compare a mutation’s residual activity with its clinical significance [12]. Pathogenicity prediction algorithms (Polyphen2_HDIV, Polyphen2_HVAR, SIFT) assess such critical variants as impairing enzyme function. Although the *c.1447G>A (p.Val483Met*) heterozygous substitution in the *ALPL* gene should be considered as a variant of unknown clinical significance, the reported clinical cases demonstrate that the *c.1447G>A (p.Val483Met*) variant alters gene expression, leading to specific clinical manifestations in the patients, and should therefore be reconsidered as likely pathogenic.

### 3.3. No Variants in the ALPL Gene

Genetic testing (the molecular analysis of the *ALPL* gene) is essential to confirm the diagnosis in the case of a clinical suspicion of HPP. The finding of pathogenic mutations in the *ALPL* gene allows a definitive HPP diagnosis to be established [16]. *ALPL* gene screening in patients with suspected HPP (based on low TNSALP levels and clinical symptoms) may be negative. This is because the genetic study of *ALPL* is limited to the sequencing of coding regions of the gene (exons), without taking into account the promoter regions or intronic regions, and large deletions [42,43]. The presence of genetic variants in other genes (*COL1A2*, *COL1A1*, *RUNX2*, etc.) in patients with suspected HPP has led to the formation of a specific panel of genes that are involved in bone fragility and muscle weakness for HPP differential diagnostics using NGS [21,30]. However, this approach does not always satisfy clinical needs. Today, studies are emerging that investigate where NGS could be used to search for genes beyond the *ALPL* that may reduce TNSALP activity or contribute to HPP symptoms [44]. Lothar Seefried et al. performed WGS for 16 patients with HPP who had TNSALP activity below the normal range. These authors observed, in four patients, other variants in the collagen, type I alpha-1 chain (*COL1A1*), nod-like receptor pyrin domain containing 12 (*NLRP12*), and sodium voltage-gated channel alpha subunit 9 (*SCN9A*), prolyl 3 hydroxylase 1 (P3H1), sarcoglycan epsilon (SGCE), and vitamin D receptor (VDR) genes [44].

We present three cases (Cases 4, 5, and 6) without detected variants in the *ALPL* gene, which required further diagnostic efforts, i.e., performing a differential diagnosis to consider alternative diagnoses. In the fourth patient, we also detected a variant in the *TRIO* gene that encodes a triple functional domain protein that functions as a guanine exchange factor and is involved in neurogenesis, cell migration, and synapse formation. Also, a variant in the *TRPV4* gene that encodes an osmo-sensitive, chemo-sensitive, and mechano-sensitive receptor involved in maintaining the Ca^2+^ influx into the cell, osteoblast differentiation, as well as peripheral nerve maturation, including the induction of neurogenesis, axon growth, and synaptogenesis was detected. In the fifth patient, we detected pathogenic biallelic variants in the *TTN* gene that are associated with Salih myopathy (Salih congenital myopathy (congenital myopathy 5 with cardiomyopathy; CMYP5; OMIM: 611705)). In the patient described in Case 6, pathogenic biallelic variants of class IV-V in the *SLC5A1* gene were associated with glucose–galactose malabsorption (GGM, OMIM: 606824), responsible for the accumulation of unabsorbed sodium, glucose, and galactose in the intestinal lumen [45]. An early presentation with diarrhea and dehydration in the neonatal period is characteristic, as well as hypernatremia and renal impairment. The identified overlapping phenotypes are presented in Table 3. The variants identified in Cases 4, 5, and 6 are associated with extremely rare diseases; hence, the awareness among doctors might be very low. However, the WES findings would allow doctors to choose an appropriate management strategy and halt the disease’s progression, as well as to improve medical and genetic counselling, disease monitoring and control, prompt treatment, and the follow-up physical and social adaptation of patients.

Today, *ALPL* gene sequencing is the first-line genetic testing for HPP, which requires clinicians to have a clear understanding of the suspected genetic cause prior to testing, underpinned by clear-cut clinical guidelines. Our study has shown that WES is not only a useful tool for identifying disease-causing genes but is also capable of correcting or changing the diagnosis. WES has a broad spectrum of advantages, such as the detection of individuals with multiple monogenic disorders and the improved characterization of blended phenotypes. The described clinical cases have shown that the presumptive diagnosis by WES can alter clinical management (by sparing additional diagnostic procedures in favor of the earlier start of treatment) for individuals and families affected by HPP. The WES analysis assists in efficiently filtering the disease-causing variants, especially single gene variants. However, the explanation of clinical findings and their interpretations based on mutations in disease-causing genes should rely on comprehensive phenotype–genotype datasets. The assessment of HPP etiology includes multiple tests, highlighting the need to develop an optimized HPP diagnostic algorithm. It should be noted that the WES is not able to detect mutations located deep in the introns. However, in rare cases, HPP can occur due to mutations of the non-coding regions of the *ALPL* gene [43]. Therefore, if the WES results are negative, but the clinical picture of HPP is typical, it may be recommended to conduct whole-genome sequencing or target sequencing of the intron regions of the *ALPL* gene.

## 4. Materials and Methods

In this study, we used Sanger sequencing to detect the heterozygous carrier status of the causal variants of the *ALPL* gene in 225 patients and homozygous carriage in 27 patients [12]. This study is a follow-up attempt to analyze the diagnostic value of confirmatory WES in 7 patients with only a single or no detected pathogenic variants in the *ALPL* gene as a result of Sanger sequencing. All of the cases have Eastern European ancestry.

### 4.1. Clinical and Biochemical Surveillances of Patients with Tentatively Diagnosed HPP

Genetic testing was performed in 7 patients with diminished blood plasma TNSALP activity [46]. The patient inclusion criteria were confirmed by a low level of *ALP* and HPP-typical clinical symptoms, Table 1.

### 4.2. Inclusion and Exclusion Criteria

Patient inclusion criteria: a low level of TNSALP activity; developmental delay (i.e., growth retardation and poor weight gain), various skeletal deformities, muscular hypotonia, and other reported HPP-based symptoms, proposed by the HPP International Working Group, Table 5 [16].

Patient exclusion criteria: multiple myeloma, milk-alkali syndrome, malnutrition, pernicious or profound anemia, profound hypothyroidism, vitamin C deficiency, Wilson’s disease, vitamin D intoxication, clofibrate therapy, starvation, Zn^2+^ or Mg^2+^ deficiency, Cushing’s syndrome, celiac disease, cardiac bypass surgery, massive transfusion, radioactive heavy metal contamination, cleidocranial dysplasia, Mseleni joint disease [47]; or the absence of informed consent for the study.

For the purpose of research, all the patients signed written informed consent forms. The investigation was performed in accordance with the Declaration of Helsinki. Our study was approved by the Academic Review Board of the D.O. Ott Research Institute of Obstetrics Gynecology and Reproductology (St. Petersburg, Russia), resolution no. 113, dated Nov. 18.

Limitations of the study: only one patient had recorded values for the natural substrates for TNSALP; the early non-traumatic loss of primary teeth is rarely described in the medical records [48].

### 4.3. Sample Preparation

Blood samples from all the patients and several of the patients’ family members were collected. The blood samples were stored for large-scale studies: #3076082 “Human Reproductive Health”. For all the blood samples, DNA was isolated through the phenol extraction method. A Quantus FluorometerTM (Promega Corporation, Madison, WI 53711, USA) and a QuantiFluor R dsDNA System (Promega Corporation, Madison, WI 53711, USA) were used to determine the DNA concentration. DNA electrophoresis in 0.6% agarose gel in a sodium borate (SB) buffer was used to assess the DNA’s integrity.

### 4.4. Sanger Sequencing of the ALPL Gene

The presence of variants identified by WES in patients was confirmed by Sanger sequencing. The analysis of the segregation of variants in the family was performed in isolated cases. The primary search for mutations in the exons of the *ALPL* gene was performed using Sanger sequencing, as described earlier [12]. The DNA was isolated by the phenol extraction method from all the blood samples. To detect the DNA concentration, we used a Quantus FluorometerTM and a QuantiFluor R dsDNA System (Promega Corporation, Madison, WI 53711, USA). To assess the DNA’s integrity, we performed DNA electrophoresis in 0.6% agarose gel in a sodium borate (SB) buffer.

### 4.5. Whole-Exome Sequencing

Whole-exome sequencing was carried out using either Illumina or the MGI sequencing platform (MGI, Beijing, China). For Illumina, we used a gDNA libraries preparation of 200 ng of gDNA, sheared to 300 bp using a Covaris S2 Focused-ultrasonicator. The fragmented DNA was transformed into DNA libraries using a KAPA Hyper Prep Kit (Roche, Washington, 98001, USA). The exome-enrichment of the DNA libraries was performed by means of a Hyper Cap Target Enrichment kit and KAPA Hyper Exome Probes set (Roche, Switzerland), according to the manufacturer’s protocol. For the MGISEQ gDNA libraries preparation, we used 200 ng of gDNA sheared to 300 bp by dint of Covaris S2 Focused-ultrasonicator. The fragmented DNA was transformed into DNA libraries by means of a KAPA Hyper Prep Kit (Roche, Switzerland) in combination with an MGIEasy DNA Adapters-96 (MGI, Beijing, China). The exome-enrichment of the DNA libraries was made using a Hyper Cap Target Enrichment kit and KAPA HyperExome Probes set (Roche, WA 98001, USA), in accordance with the manufacturer’s protocol with the modifications: 1 uL of Block3 and 10uL of Block4 reagents from the MGIEasy Exome Capture Accessory kit were supplemented to the hybridization mix in lieu of KAPA Universal Enhancing Oligos, and the final library amplification was made by means of an MGI PCR Primer Mix. For the preparation of the DNA libraries, we used an MGIEasy Circularization Module V2.0 (MGI, Beijing, China). Library quantitation was carried out by a Quantus Fluorometer with a QuantiFluor^®^ dsDNA System kit (Promega Corporation, Madison, WI 53711, USA). A high-sensitivity DNA assay with gel electrophoresis using the 2100 Bioanalyzer System (Agilent Technologies, Santa Clara, CA 95051, USA) was conducted for accurate DNA size determination and quality control (between 300 to 400 bp). Paired-end reads of no shorter than 100 bp were generated for each sample.

### 4.6. Bioinformatic Data Analysis and Variant Calling in Patient Exomes

All the exome samples were matched onto a GRCh38.p13 reference genome assembly by using the Genome Analysis ToolKit (GATK) [49] and the BWA MEM read aligner [50]. Next, the search for genetic variants was performed using the GATK HaplotypeCaller v. 4.1.4, followed by the genotyping of the samples [51]. Next, the genetic variants were filtered by means of GATK. All genotypes with a total read depth of less than 10 were excluded. The filtered genetic variants were annotated using the Ensembl Variant Effect Predictor (VEP) v103.1, based on the 1000 Genomes, phase 3 [52], exome aggregation consortium [53], in-house Russian exome allele frequencies [54,55], as well as the NCBI ClinVar and dbNSFP v 2.9 [56] and the *ALPL* mutation database [57]. Custom software (v 4.0) was used for enhanced variant interpretation. The sequencing data were subjected to bioinformatic analysis, pathogenicity prediction (with SIFT, Polyphen2_HDIV, Polyphen2_HVAR, etc.), a clinical symptom comparison/pedigree analysis, a database search, and a literature review for relevant diseases. The pathogenicity of the identified genetic variants was assessed in accordance with the ACMG guidelines (2015) [48].

### 4.7. Molecular Docking of TNSALP

The structure of the dimeric TNSALP complex was obtained from the PDB database (7YIX); the structure of the WNT10A protein was downloaded from the AlphaFold Protein Structure Database, since the structure of this protein has not been experimentally determined (AF-Q9GZT5-F1-v4). The structures of proteins with inserted mutations were obtained using the homology modeling method in the MODELLER tool, where the downloaded structures were used as templates. The obtained structures (wild-type and mutated) were solvated in water in a triclinic box with added ions. The resulting systems were minimized using steepest-descent over 50,000 steps. The water around the protein structures was then equilibrated in two phases (in NVT ensemble, then in NPT ensemble) for 50,000 steps (dt = 2 fs). Molecular dynamics simulations were performed for 250,000,000 steps (dt = 2 fs)—500 ns. From the molecular dynamics results, the atomic standard deviation and the RMS fluctuation of amino acid residues were obtained using the GROMACS software package (2022.5 version).

## 5. Conclusions

The presented cases demonstrate a wide variability in HPP-associated clinical manifestations that does not lend itself to an explanation based on the findings of the *ALPL* gene sequencing alone. In patients with HPP-typical symptoms, WES provides extra insights, allowing the confirmation of a diagnosis or possibly yielding evidence that is suggestive of a completely different diagnosis in some cases. HPP comorbidity is a most complicated challenge for diagnoses. In such cases, whole genomic sequencing (WGS) or WES can significantly reduce the time between referral and a diagnosis and provide patients with earlier and more optimal treatment. Given that the frequency of HPP-based clinical manifestations may vary across different populations [12,32] and the clinical presentation is often atypical, efforts are needed to develop an algorithm for HPP differential diagnostics.

## Figures and Tables

**Table 1:** Clinical symptoms and genetic variants in HPP patients.

Symptoms	Patient 1	Patient 2	Patient 3	Patient 4	Patient 5	Patient 6	Patient 7
	Obligate diagnostic criterion for HPP in children						
ALP level in serum 1 (age)	1 year 8 months 38 IU/L (reference 150–370 IU/L)	3.5 years 119 IU/L (reference 180–720 IU/L)	14 years 43 IU/L (reference 160–500 IU/L)	1 year 8 months 85 IU/L (reference 150–370 IU/L)	3 months 70 IU/L (reference 70–350 IU/L)	1 year 1 month 139 IU/L (reference 150–370 IU/L)	3 years 9 months 126 IU/L, (reference 125–320 IU/L)
ALP level in serum 2 (age)	5 years 69–73 IU/L (reference 180–720 IU/L)	4 years 120.3 IU/L (reference 180–720 IU/L)	14.5 years 81 IU/L (reference 160–500 IU/L)	-	8.5 months 233.2 IU/L (reference 70–350 IU/L)	4 years 6 months 118 IU/L (reference 180–720 IU/L)	-
	Major diagnostic criteria for HPP in children						
WES results: variants in *ALPL* gene	c.1447G>A in exon 12 of ALPL gene (chr1:g.21904013G>A; rs1256212515), heterozygous (78x)	c.1447G>A in exon 12 of *ALPL* gene (chr1:g.21904013G>A; rs1256212515), heterozygous (59x)	c.205G>A in exon 4 of *ALPL* gene (chr1:21887613G>A; rs1178008018), heterozygous (31x)	Not detected	Not detected	Not detected	Not detected
Elevation of natural substrates of TNSALP	Yes	No data	No data	No data	No data	No data	No data
Early non-traumatic loss of primary teeth	No data	Yes	No	No data	No data	Yes	Yes
	Minor diagnostic criteria for HPP in children						
Skeletal deformities	Funnel chest Skull deformities Hydrocephalus	Planovalgus foot deformity	No	Incomplete ossification of parietal bones, open sagittal suture, chest deformity	Funnel chest	Valgus Knee, valgus foot deformity, chest deformity, hyperkyphosis	Valgus foot deformity, chest asymmetry
Short stature	No data	Yes	No	Yes	No	Yes	Yes
Height/Weight/Age	No data/14.5 kg (10 years) ▼	94 sm/14.5 kg (4 years) ▼	172 sm/91 kg (14 years) ▲	69 sm/5 kg (2 years) ▼	72 sm/9.3 kg (1 year) Normal	95 sm/14.5 kg (4.5 years) ▼	74 sm/5.86 kg (3.9 years) ▼
Limb shortening	No	No	No data	Yes	No	Yes	No data
Gait disturbance	Unable to walk	No	No	Unable to walk	Unable to walk (young age)	No data	No data
Joint pathology	No	No	Pain in the left knee joint No data	Contracture in the right hand thumb, stiffness of knee and ankle joints	Contractures of wrist joints	No data	No data
Muscular hypotension	Yes	Yes	No data	Yes	Yes	Yes	Yes
Delayed motor development	Yes	Yes	No	Yes	Yes	Yes	Yes
Neurological symptoms	Mental and speech delay	No	Tension headaches	Mental and speech delay	Signs of peripheral polyneuropathy by electroneuromyography	Perinatal hypoxic- ischemic damage, mental and speech delay	Mental and speech delay
Digestive disorders, malnutrition	Low weight, frequent regurgitation in infancy	Low weight, constipation	High weight	Low weight	No	Severe infectious gastroenterocolitis, hypotrophy, malabsorption	Low weight
Fractures	Yes	No	No	No	No	No data	No
Poorly healed fractures	No data	No	No	No	No	No data	No
Hypomineralization of bone tissue/osteoporosis	Yes	Yes	Yes	No data	No	No	No
Respiratory failure	No	Yes	No	No data	No	No data	No
Hypercalcemia	No	No	No	No	No	No	No data
Hypercalciuria	No	No	No	No	No	No data	No data
Hyperphosphatemia	No	Yes	No	No	No	No data	No data
Nephrocalcinosis/kidney damage	Pyeloectasia of kidneys	No	No data	No data	No	Pyeloectasia of the right kidney, calcenates	Pyeloectasia of the right kidney, hydrocalicosis with incomplete bilateral doubling of the renal pelvis
Convulsions	Pharmaco- resistant epilepsy	No	No	No data	No	No data	No
Multiple organ failure	No	No	No	No	No	Yes	No
Visual disorders	Myopia, astigmatism	Hypermetropia, astigmatism	Micropsia	No	No	No data	Myopia, strabismus
Hair features	No	Brittle hair	Hair loss	No	No	No data	No data
Preliminary diagnosis	HPP? Focal epilepsy	HPP?	HPP?	HPP? Protein-energy malnutrition	HPP?	HPP?	HPP? Protein-energy malnutrition.
WES results: variants in other genes	c.3470del in exon 23 of *SMC1A* gene (chrX:g.53407975CT>C), heterozygous (30x)	c.682T>A in exon 3 of *WNT10A* gene(chr2:g.219755011T>A; rs121908120), homozygous (67x)	Not detected	c.5651A>C in exon 36 of *TRIO* gene(chr5:g.14463018A>C), heterozygous (45x); c.880T>G ʙ exon 6 of *TRPV4* gene (chr12:g.110236691A>C), heterozygous (68x)	c.32078-1G>T in intron 159 of *TTN* gene (chr2:g.179516477C>A), heterozygous (100x); c.47720_47721del in exon 235 of *TTN* gene (chr2:g.179466391CTT>C), heterozygous (121x)	c.1946G>A in exon 15 of *SLC5A1* gene (chr22:g.32506151G>A), homozygous (125x)	Not detected
Post-WES diagnosis	HPP infantile. Developmental and epileptic encephalopathy 85, with or without midline brain defects (DEE85)	HPP childhood	HPP childhood	Hereditary motor and sensory neuropathy, type IIc; mental developmental disorder, autosomal dominant 63, with macrocephaly (OMIM: 618825)?	Hereditary neuromuscular disease: congenital myopathy 5 with cardiomyopathy; CMYP5(OMIM: 611705) Muscular dystrophy, limb–girdle, autosomal recessive 10?	Glucose–galactose malabsorption	Fetal alcohol syndrome
Diagnostic and management tactics	Treatment of comorbid conditions	Further observation, HPP treatment	HPP treatment	Additional examination	Additional examination	Additional examination	Symptomatic treatment

Note ▲, ▼ increase, decrease in height and body weight compared to the norm.

**Table 2:** The genetic variants identified in HPP patients.

Case No.	Gene	Protein	Gene-Associated Diseases	Variants	Mutation Type	AF (gnomAD)	AF (RUSeq)	Pathogenicity
1, 2	*ALPL*	Alkaline phosphatase, biomineralization associated	OMIM: 146300, 241500, 241510	c.1447G>A in exon 12 (chr1:g.21904013G>A; rs1256212515)	missens	0.00001971 *	0.0002966 *	Likely pathogenic (IV)
3				c.205G>A in exon 4 (chr1:21887613G>A; rs1178008018)	missens	N/A	N/A	Uncertain significance
1	*SMC1A*	Structural maintenance of chromosomes 1A	OMIM: 300590; 301044	c.3470del in exon 23 (chrX:g.53407975CT>C)	frameshift	N/A	N/A	Pathogenic (V)/VUS
2	*WNT10A*	Wnt family member 10A	OMIM: 257980; 224750; 150400	c.682T>A in exon 3 (chr2:g.219755011T>A; rs121908120)	missens	0.01405 *	0.01130	Conflicting interpretations of pathogenicity
4	*TRIO*	Trio Rho guanine nucleotide exchange factor	OMIM: 617061; 618825	c.5651A>C in 36 exon (chr5:g.14463018A>C)	missens	N/A	N/A	Uncertain significance (VUS, III)
4	*TRPV4*	Transient receptor potential cation channel subfamily V member 4	OMIM: 606071	c.880T>G ʙ exon 6 (chr12:g.110236691A>C)	missens	N/A	N/A	Uncertain significance (VUS, III)
5	*TTN*	Titin	OMIM:611705; 608807	c.32078-1G>T in intron 159(chr2:g.179516477C>A)	splicing site	N/A	N/A	Likely pathogenic (IV)
				c.47720_47721del in exon 235 (chr2:g.179466391CTT>C)	Frameshift	N/A	N/A	Likely pathogenic (IV)
6	*SLC5A1*	Solute carrier family 5 member 1	OMIM: 606824	c.1946G>A in exon 15 (chr22:g.32506151G>A)	missens	0.00000657 *	N/A	Likely pathogenic (IV)

Note * at the time of the survey there were no frequency data. N/A—not applicable

**Table 3:** Differential diagnostics of patients with primary HPP diagnosis and WES findings (cases 1, 2, 4, 5, 6).

Case	Sex	Age; Years	Age of HPP Diagnosis	Features Consistent with HPP	Detected Genetic Variants		An Alternative Diagnosis	Features Typical for an Alternative Diagnosis and Not Typical for HPP	Phenotypic Overlaps
					1st Molecular Mechanism (Variants in *ALPL* Gene)	2nd Molecular Mechanism (Variants in Other Genes)			
1	f	10	6	↓ ALP in serum; ↑ Pi and PEA; muscle hypotonia; epileptic paroxysms; rickets-like bone deformities; osteoporosis; developmental delay	c.1447G>A (chr1:g.21904013G>A; rs1256212515); heterozygous	c.3470del in *SMC1A* gene (chrX:g.53407975CT>C; heterozygous	Developmental and epileptic encephalopathy type 85 (DEE85; OMIM# 301044)	Refractory seizures in the first year of life; global developmental delay with impaired intellectual development and poor or absent speech; dysmorphic features.	Epileptic paroxysms; developmental delay
2	f	4	4	↓ ALP in serum; muscle hypotonia; short stature; developmental delay; respiratory attacks; brittle hair; tooth decay	c.1447G>A (chr1:g.21904013G>A; rs1256212515); heterozygous	c.682T>A in *WNT10A* gene (chr2:g.219755011T>A; rs121908120); homozygous	Ectodermal dysplasias: odontoonychodermal dysplasia (OMIM: 257980); Schopf-Schulz-Passarge syndrome (OMIM: 224750); tooth agenesis; selective; 4 (OMIM: 150400)	Ectodermal pathology; tooth agenesis	↓ ALP in serum; brittle hair and tooth decay
4	m	2	2	↓ ALP in serum; ↓ionized calcium skeletal deformities; muscle hypotonia; short stature; limb shortening; joint pathology; developmental delay	no	c.5651A>C in exon 36 of *TRIO* gene (chr5:g.14463018A>C; heterozygous; c.880T>G ʙ exon 6 of *TRPV4* gene (chr12:g.110236691A>C; heterozygous	Hereditary motor and sensory neuropathy; type IIc; Mental developmental disorder; autosomal dominant 63; with macrocephaly (OMIM: 618825)	Dysmorphic features: deformed auricles, sunken nose bridge, microretrogenia	↓ ALP in serum(?); Skeletal deformities; muscle hypotonia; short stature; limb shortening; joint pathology; developmental delay
5	f	1	0	↓ ALP in serum; muscle hypotonia; skeletal deformities (funnel chest); joint pathology; developmental delay	no	c.32078-1G>T (chr2:g.179516477C>A); heterozygous; c.47720_47721del (chr2:g.179466391CTT>C); heterozygous in *TTN* gene	Congenital myopathy 5 with cardiomyopathy; CMYP5 (OMIM: 611705 Muscular dystrophy; limb-girdle; autosomal recessive 10	↑Creatinine phosphate kinase; polyneuropathy; early disease onset; contractures	Skeletal deformities; developmental delay
6	f	4	1	↓ ALP in serum; muscle hypotonia; short stature; skeletal deformities; limb shortening; developmental delay; urolithiasis; gastroenteritis; early loss of primary teeth	no	c.1946G>A in *SLC5A1* gene (chr22:g.32506151G>A); homozygous	Glucose-galactose malabsorption (OMIM: 606824)	Born in a consanguineous marriage; sibling malabsorption syndrome; diarrhea; hypokalemia; hyponatremia	↓ ALP in serum; Developmental delay; muscular hypotonia; skeletal deformities; urolithiasis

Note ↓ decrease in concentration of TNSALP, ↑ increase in concentration.

**Table 4:** Overview of different differential diagnoses for decreased TSALP activity.

Drugs	Anti-Resorptive Therapy, Chemotherapy, Excess Vitamin D
Endocrine disorders	Hypoparathyroidism Hypothyroidism Hypercortisolism Renal osteodystrophy and adynamic bone disease Delayed growth and puberty
Hematological conditions	Pernicious anemia Massive blood transfusions Myeloproliferative disorders Myeloma
Nutritional deficiencies	Magnesium Zinc Vitamin C B6, B12, and folate Protein/calorie Copper Celiac disease Milk-alkali syndrome Starvation
Miscellaneous	Severe illness Major surgery or trauma Wilson’s disease Achondroplasia Osteogenesis imperfecta Cleidocranial dysplasia Mseleni joint disease Disorders affecting linear growth in childhood

**Table 5:** Recommended criteria for HPP diagnosis in children.

Obligate Diagnostic Criterion	Major Diagnostic Criteria for	Minor Diagnostic Criteria
Low TNSALP enzymatic activity for age and sex	*ALPL* gene variant(s) Elevation of natural substrates of TNSALP Early non-traumatic loss of primary teeth Presence of rickets on radiography	Short stature Delayed motor milestones Chronic musculoskeletal pain Impaired mobility Genu valgum/varum Craniosynostosis Nephrocalcinosis/nephrolithiasis Low muscle tone

## Data Availability

All the data used for the analyses in this report are available from the corresponding author upon reasonable request.
